# Physical Properties, Antioxidant and Antimicrobial Activity of Chitosan Films Containing Carvacrol and Pomegranate Peel Extract

**DOI:** 10.3390/molecules200611034

**Published:** 2015-06-15

**Authors:** Gaofeng Yuan, Hua Lv, Bingjie Yang, Xiaoe Chen, Haiyan Sun

**Affiliations:** 1 Key Laboratory of Health Risk Factors for Seafood of Zhejiang Province, Zhejiang Ocean University, Zhoushan 316022, China; E-Mails: yuangf@zjou.edu.cn (G.Y.); Xiaoechen@163.com (X.C.); 2 College of Food and Medicine, Zhejiang Ocean University, Zhoushan, Zhejiang 316022, China; E-Mails: climbery@126.com (H.L.); 18268732445@163.com (B.Y.)

**Keywords:** chitosan film, antioxidant activity, antibacterial activity, pomegranate peel extract, carvacrol

## Abstract

Chitosan-based active films were developed by incorporation of carvacrol (10 g/L), pomegranate peel extract (PPE, 10 g/L) and carvacrol + PPE (10 g/L of each) and their physical, antioxidant and antimicrobial properties were investigated. Incorporation of carvacrol and carvacrol + PPE into the films significantly decreased the water vapor permeability, tensile strength and percentage of elongation at break. Incorporation of carvacrol, PPE and carvacrol + PPE into the films decreased the transparency, but significantly increased the total phenol content and antioxidant activity. All the films, with the exception of PPE-incorporated film, exhibited antibacterial activity against *Escherichia coli* and *Staphylococcus aureus*. In addition, the antibacterial activity against *Staphylococcus aureus* of the film incorporated with carvacrol + PPE was moderately higher than that incorporated with carvacrol or PPE alone, suggesting a synergistic action between carvacrol and PPE.

## 1. Introduction

Packaging plays an important role in the conservation, distribution and marketing of foodstuffs. Studies within the area of food active packaging are experiencing a great development due to consumer demand and market trends. Currently, there is an interest in films produced from natural resources due to the excellent biodegradability, biocompatibility, edibility and their potential applications. These films may operate as carriers of many functional ingredients that may include preservation agents (antimicrobial/antioxidant (AA) agents), flavors, spices and colorants which improve the functionality of the packaging materials by adding novel or extra functions [1].

Chitosan, a cationic polysaccharide consisting of (1,4)-linked-2-aminodeoxy-β-d-glucan, is the deacetylated form of chitin. Chitosan, which has attracted attention as a potential food preservative of natural origin, has been classified as Generally Recognized as Safe (GARS) by the US Food and Drug Administration (FDA) in 2001 [2]. Chitosan is considered as an ideal biopolymer for the production of active edible films due to its non-toxicity, biocompatibility, biodegradability and film-forming ability [3]. Although chitosan is a promising biopolymer for food active packaging, it has no significant antioxidant [4] and ambiguous antimicrobial activity [5,6], so improvement of the antioxidant and antimicrobial activity of chitosan could expand its applications in food active packaging. One strategy for this is to incorporate AA agents into a chitosan film to improve food safety and shelf life [7]. In terms of active agents that can be incorporated into films, plant extracts have received much attention as they contain high concentrations of phenolic compounds that possess strong antioxidant activity. As a result, special attention has been focused on those available from inexpensive or residual sources from the agriculture industries [8].

It has been reported that pomegranate peel and pomegranate peel extract (PPE) have significant free radical scavenging, anti-microbial, antiatherogenic and antimutagenic properties [9]. These findings have led to increased interest in PPE. Active films incorporated with PPE were already developed [8,10]. Carvacrol is a phenolic compound extracted from oregano and thyme oil that possesses antimicrobial and antioxidant properties, and a particular aroma which makes it an attractive ingredient for certain types of foods [11,12]. Carvacrol is generally considered safe for consumption and is in the category of food additives permitted for direct addition in human food (US FDA). The use of combinations of natural antimicrobial (AM) agents may increase the spectrum of AM activity and also produce synergistic interactions against microorganisms [13]. The objectives of the present study were to develop composite films from chitosan incorporated with carvacrol (10 g/L) and PPE (10 g/L) alone and in combination, and to investigate the effect of these extracts on the physical, mechanical, antimicrobial and antioxidant properties of the resulting films.

## 2. Results and Discussion

### 2.1. Film Thickness, Color and Opacity

The effects of carvacrol and PPE incorporation on film thickness color and opacity are shown in Table 1. The results showed that the thickness of the four films varied between 0.090 and 0.126 mm and incorporation of 10 g/L carvacrol, 10 g/L PPE and 10 g/L carvacrol + 10 g/L PPE into the films had no significant difference in thickness.

**Table 1:** Color value and opacity for chitosan films incorporated with pomegranate peel extract and carvacrol.

Film Samples	Thickness (mm)	Opacity (A·mm^−1^)	Color		
			L*	a*	b*
Control	0.105 ± 0.12a	1.290 ± 0.19	90.43 ± 0.26a	−0.67 ± 0.41a	11.93 ± 0.28c
CR	0.091 ± 0.23a	1.411 ± 0.37b	91.23 ± 0.44a	−0.18 ± 0.45a	8.03 ± 0.49d
PPE	0.111 ± 0.21a	2.394 ± 0.42a	63.30 ± 0.22b	14.30 ± 0.32b	54.60 ± 0.62a
CR + PPE	0.126 ± 0.34a	2.098 ± 0.32a	57.50 ± 0.17b	18.00 ± 0.29c	51.20 ± 0.56b

Mean values in each column with different lower case letters are significantly different (*p* < 0.05). PPE, pomegranate peel extract; CR, carvacrol.

Incorporation of carvacrol into chitosan films significantly decreased b* values (yellowness/blueness) of the film, compared with the control (*p* < 0.05). This finding is consistent with that of López-Mata *et al.* [11], who found that chitosan films with 0.5%, 1.0% and 1.5% *v*/*v* of carvacrol showed a tendency to yellow (b*). Incorporation of PPE alone and in combination with carvacrol into chitosan films also significantly affected L* (lightness/darkness), a* (redness/greenness) and b* values of the films, compared with the control (*p* < 0.05). PPE and PPE + carvacrol incorporation into chitosan films leads to a decrease of L* and an increase of a* parameters, indicating a decrease of the lightness and an increase of the redness of the films when compared with the control. The decrease tendency of lightness in the PPE-incorporated film could be attributed to the presence of polyphenols in PPE. Similar results have been observed where chitosan films incorporated with aqueous green tea extract and grape seed extract containing polyphenols decreased the film’s lightness [4,14,15]. This could be a drawback for the increasing demand for transparent and colorless food packaging materials [16]. However, the decrease in the film’s lightness may help avoid oxidative deterioration in packaged foods caused by exposure to visible and ultraviolet light, leading to nutrient losses, discoloration and off-flavors [14].

The transparency of films incorporated with 10 g/L carvacrol was slightly decreased, which is consistent with the results of Lopez-Mata *et al*. [11], who also found that the transparency of chitosan films with 1.0% and 1.5% *v*/*v* of carvacrol was decreased. On the other hand, the transparency of films incorporated with 10 g/L PPE and 10 g/L carvacrol + 10 g/L PPE was dramatically decreased when compared with the control (Table 1). This finding is agreement with those of Qin *et al*. [8], who observed that the transparency of chitosan films incorporated with 1%, 1.5%, and 2% *w*/*v* of pomegranate rind extract showed a marked reduction. The change in transparency may be caused by the presence of polyphenols in films.

### 2.2. Water Vapor Permeability

**Figure 1:**
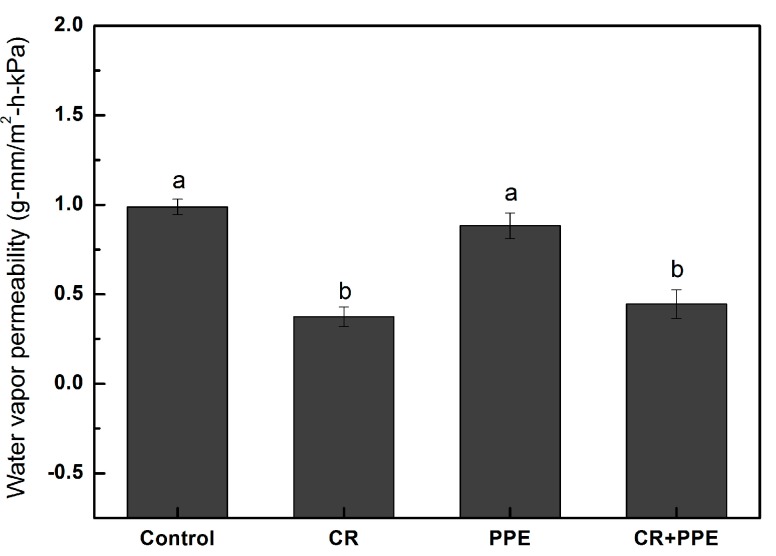
Water vapor permeability for chitosan films incorporated with pomegranate peel extract and carvacrol. Each data is the mean values per treatment and time point (mean ± standard error). Values not sharing a lower case letter are significantly different at *p* < 0.05. PPE, pomegranate peel extract; CR, carvacrol.

The water vapor permeability (WVP) of the films is shown in Figure 1. Incorporation of 10 g/L PPE alone into the film had no significant difference in WVP. However, incorporation of 10 g/L carvacrol and 10 g/L carvacrol + 10 g/L PPE into the films significantly decreased the WVP when compared with the control.

Chitosan films have been proven to present moderate oxygen barrier properties and good carbon dioxide barrier properties but high permeabilities to water vapor, due to their hydrophilic nature [17]. In order to improve water barrier properties of chitosan films, hydrophobic compounds, such as lipids and essential oils are frequently incorporated [18,19]. Carvacrol, a major constituent of oregano essential oil has been added to chitosan films and has been proved to decrease the WVP of chitosan films [14]. The present study confirmed this finding. The decrease in WVP in carvacrol-incorporated chitosan films could be due to modification of the hydrophobic portion of the film, as a result of the ratio between chitosan and carvacrol [20].

### 2.3. Mechanical Properties

The mechanical properties of films are presented in Table 2. Compared with the control, incorporation of 10 g/L PPE alone showed no significant difference on the tensile strength (TS) and percentage elongation at break (E%) of the chitosan films. This finding is contradictory with those of Siripatrawan and Harte [15] who found that addition with green tea extract into films significantly increased the TS and E%, probably due to the interaction between chitosan matrix and polyphenolic compounds from green tea extract.

**Table 2:** Tensile strength and percentage elongation at break for chitosan films incorporated with pomegranate peel extract and carvacrol.

Film Samples	Tensile (MPa)	E%
Control	22.23 ± 2.02a	31.51 ± 3.48a
CR	8.54 ± 1.43c	17.37 ± 2.96b
PPE	23.50 ± 2.48a	30.76 ± 4.14a
CR + PPE	15.91 ± 2.87b	21.67 ± 2.88b

Mean values in each column with different lower case letters are significantly different (*p* < 0.05). PPE, pomegranate peel extract; CR, carvacrol.

On the other hand, incorporation of 10 g/L carvacrol alone and in combination with PPE showed significant decrease on the TS and E% of the chitosan films. Similar results were observed in chitosan films with added carvacrol [11,14], α-tocopherol [21] and *Zataria multiflora* Boiss essential oil [7]. The change in mechanical properties could be due to the development of a structure with less mobility and therefore less flexibility and resistance to fracture after the addition of hydrophobic agents to the film composition [22].

### 2.4. Total Phenolics Content and Antioxidant Activity

**Figure 2:**
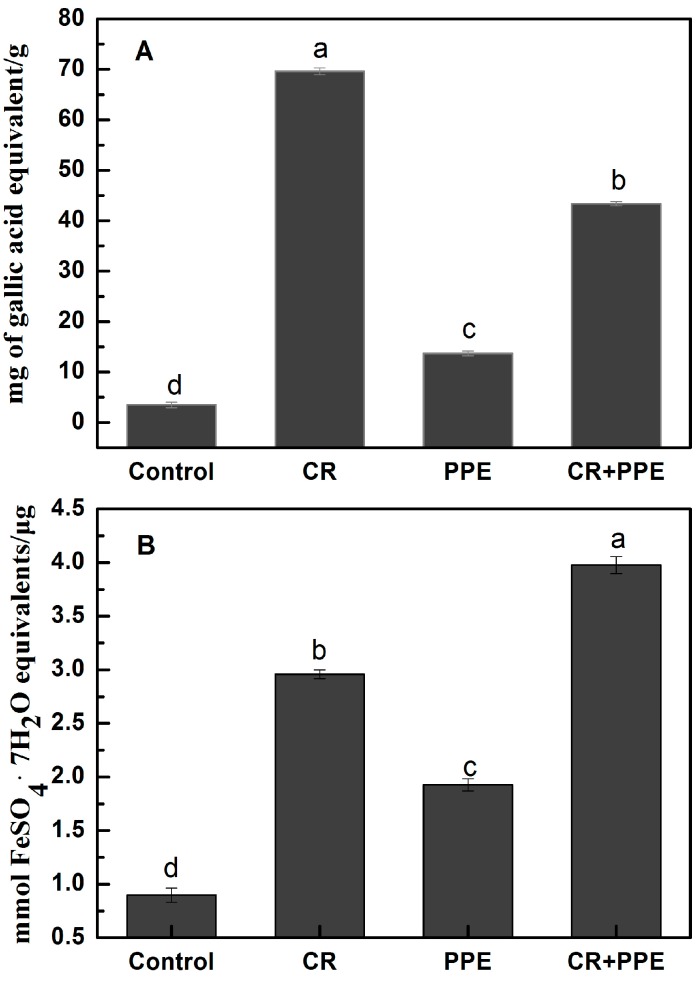
Total phenol contents (**A**) and antioxidant activity (**B**) for chitosan films incorporated with pomegranate peel extract and carvacrol. Each data is the mean values per treatment and time point (mean ± standard error). Values not sharing a lower case letter are significantly different at *p* < 0.05. PPE, pomegranate peel extract; CR, carvacrol.

Compared with the control, the antioxidant activity of the films was significantly increased when incorporated with 10 g/L PPE, 10 g/L carvacrol and 10 g/L carvacrol + 10 g/L PPE (Figure 2). Similar studies have found that carvacrol and its derivatives have considerable antioxidant activity and addition of carvacrol to the chitosan film could produce antioxidant activity [11,23]. The antioxidant capacity of carvacrol depends on the steric and electronic effect of its ring, besides the presence of the hydroxyl group, which is capable of donating hydrogen atoms [23]. Other studies have described that the antioxidant activity of the chitosan film was dramatically increased after incorporating PPE in the film [8]. Kanatt *et al*. [10] also found that the antioxidant activity was significantly increased in chitosan-polyvinyl alcohol film incorporated with PPE. The present study matches those findings that indicated that PPE conferred antioxidant properties to active films. 

According to the studies by Gomez-Estaca *et al.* [24] and Moradi *et al.* [7], the degree of antioxidant power of edible film is generally proportional to the amount of antioxidant additives added, so it was not unexpected that the antioxidant activity of the film incorporated with 10 g/L carvacrol + 10 g/L PPE was significantly higher than that incorporated with 10 g/L carvacrol or 10 g/L PPE alone, suggesting an additive effect between carvacrol and PPE. 

### 2.5. Antibacterial Activity

The antibacterial activity of the films is shown in Table 3. The results showed that the chitosan films incorporated with 10 g/L carvacrol and 10 g/L carvacrol + 10 g/L PPE have significantly inhibitory activity against Gram-negative *E. coli*, and those incorporated with 10 g/L PPE, 10 g/L carvacrol and 10 g/L carvacrol + 10 g/L PPE also have significantly inhibitory activity against Gram-positive *S. aureus*.

**Table 3:** Antibacterial activity expressed as the inhibition zone diameter (mm) of chitosan films incorporated with pomegranate peel extract and carvacrol.

Film Samples	Inhibition Zone (mm)	
	*S. aureus*	*E. coli*
Control	ND	ND
CR	12.33 ± 0.33b	11.33 ± 0.33a
PPE	2.50 ± 0.29c	ND
CR+PPE	16.50 ± 0.29a	11.67 ± 0.88a

Mean values in each column with different lower case letters are significantly different (*p* < 0.05). PPE, pomegranate peel extract; CR, carvacrol; ND, not detected.

Several studies have shown that PPE exhibited antimicrobial activity both *in vitro* (agar diffusion) and *in situ* (in food) [25,26,27]. Incorporation of 10 g/L PPE into films showed significant antibacterial activity against *S. aureus*, however no antibacterial activity against *E. coli* was revealed in the present study. In general Gram-positive bacteria are considered more sensitive than Gram-negative bacteria to antimicrobial compounds [28]. This is generally attributed to the differences in the structure of their cell walls, as the cell walls of Gram-negative bacteria contain lipopolysaccharides, which may prevent active components from reaching the cytoplasmic membrane [28,29]. The present results were in agreement with previous studies in which Gram negative bacteria seemed to present higher resistance against PPE-incorporated chitosan-polyvinyl alcohol film and grape seed extract-incorporated carrageenan film [10,30].

The inhibitory effect of carvacrol on the growth of various microorganisms has been studied extensively and is well documented [12]. The antimicrobial efficiency of carvacrol-incorporated film against *E. coli* was previously reported in low density polyethylene (LDPE)/polyamide films [31] and in wheat gluten montmorillonite-coated papers [32]. The inhibitory effect of carvacrol-incorporated film against both *E. coli* and *S. aureus* was also shown in polyethylene-co-vinylacetate containing carvacrol [33], and sodium and calcium caseinate films with carvacrol [16]. Incorporation of 10 g/L carvacrol into chitosan films showed significant antibacterial activity against *S. aureus* and *E. co*li in the present study, which matches the previous studies [16]. The antibacterial mechanism of carvacrol could due to the interaction with lipophilic components of the bacterial membrane, which can cause changes in the permeability of H^+^ and K^+^, and finally damage the essential functions and cause cell death [34].

In addition, the antibacterial activity against the *S. aureus* in the film incorporated with 10 g/L carvacrol + 10 g/L PPE was significantly higher than that incorporated with 10 g/L carvacrol or 10 g/L PPE alone, suggesting that there is a synergistic action between carvacrol and PPE. As a general conclusion, the incorporation of carvacrol and PPE to chitosan films conferred some antimicrobial activity against bacterial strains potentially present in food to the films.

## 3. Experimental Section

### 3.1. Materials

The bacterial strains used in this study were *Escherichia coli* ATCC8099 and *Staphylococcus aureus* ATCC6538. Chitosan with degree of deacetylation of 90% were purchased from Shanghai Jinsui Biotechnology Company (Shanghai, China). The dried pomegranate peel was powdered using a mixer grinder and two hundred gram portions of finely-powdered pomegranate peel was blended with 80% methanol for 2 h at 40 °C in a shaking water bath. The ratio pomegranate peel powder: solvent was 1:10 (*w*/*v*). The extracts were through filter paper (Waterman No. 1) and concentrated under vacuum with a rotary evaporator (Eyela, Rikakikai Co., Ltd., Tokyo, Japan). The concentrate was dried overnight in an oven at 40 °C to form powder which was stored at 4 °C until further analysis.

### 3.2. Film Preparation

Chitosan solution was prepared with 2% (*w*/*w*) chitosan in 1% (*w*/*w*) acetic acid at room temperature. After overnight agitation, the solution was filtered using a filter cloth to remove any insoluble particles. Afterward, glycerol (glycerol/chitosan = 0.5, *w*/*w*) and Tween 80 at 0.5% (*w*/*w*) were mixed into the solution, with 30 min of stirring. Then, PPE and carvacrol were added to the chitosan solution. The following four solutions were prepared: (i) control, without added agents; (ii) chitosan with 10 g/L carvacrol; (iii) chitosan with 10 g/L PPE; (iv) chitosan with 10 g/L carvacrol + 10 g/L PPE. After addition of PPE and carvacrol, all the solutions were homogenized at 13,000 rpm for 5 min to obtain an emulsion. Chitosan films were prepared by casting/solvent evaporation method. The emulsions were poured into glass Petri dishes dried at incubator chamber (25 ± 2 °C) for 48 h with 50% ± 2% relative humidity (RH). The films were then peeled from the plates and placed at 50% ± 2% RH at 25 °C.

### 3.3. Film Thickness

Film thickness was measured using a micrometer (Mitutoyo Corporation, Kanagawa, Japan) at five different locations of the film and the mean thickness was used to calculate the film properties.

### 3.4. Film Color and Opacity Measurements 

The color of film samples was measured by using an automatic color difference meter (DC-P3, Shanghai Go On Chemical Co., Ltd., Shanghai, China). L* (lightness) represents the brightness on a scale of 0 (dark) to 100 (white), a* (redness) scale ranges from negative values for green to positive values for red and b* (yellowness) scale ranges from negative values for blue to positive values for yellow. The colorimeter was calibrated with a certified standard white plate. Film pieces (20 mm in diameter) were evenly distributed on a white reflector standard plate as background and L*, a* and b* values were measured with a halogen lamp as an illuminant. For each sample, six measurements were taken on each.

Opacity was determined by measuring the film absorbance at 600 nm using a UV spectrophotometer (UV-2800, Unico, New York, NY, USA). The films were cut into rectangular shapes and directly placed on the internal side of the spectrophotometer cell. An empty test cell was used as the reference. The opacity of the films was calculated by the following equation:

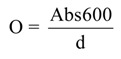
(1)
where O is the opacity, Abs600 is the value of absorbance at 600 nm and d is the film thickness (mm).

### 3.5. Water Vapor Permeability

WVP was determined following ASTM method with slight modification [35]. Film samples were sealed to glass cups having 5 cm diameter containing water. Film-covered cups were placed in an environmental chamber set at 25 °C and 50% RH. The weight of cup was determined periodically until steady state was reached. Water vapor transmission rate (WVTR) of the films was determined from the slope of the weight change of the cup *vs.* time curve. The WVTR of the films was then used to calculate the WVP using the following equation:

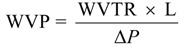
(2)
where WVTR was the measured water vapor transmission rate (g/(m^2^ s)), L is the mean film thickness, and Δ*P* is the partial water vapor pressure difference (Pa) across the two sides of the film.

### 3.6. Mechanical Properties

TS and E% were measured with a Universal Testing Machine (TA.XT plus model, Stable Micro Systems, Surrey, UK) fitted with a 50 N static load cell. The films were cut into strips of 25 mm wide and 100 mm long. Initial grip separation and cross-head speed were set at 50 mm and 5.0 mm/s, respectively. TS and E% was evaluated in six samples from each type of film.

### 3.7. Total Phenolic Content Assay

Films (25 mg) were homogenized with ethanol during 5 min at 10,000 rpm and then centrifuged at 3000 rpm during 10 min. The extracts were used to determine the total phenolic content and antioxidant activity. Phenolic compounds were determined using Folin-Ciocalteu reagent method by reading the absorbance at 765 nm with a UV-Vis spectrophotometer (UV-2800, New York, NY,USA) [36]. Gallic acid was used as a standard and the results were expressed as milligrams of gallic acid equivalent (GAE)/grams of film.

### 3.8. Antioxidant Activity

Ferric reducing antioxidant power (FRAP) assay was determined according to the method of Benzie *et al.* [37]. The working FRAP reagent was prepared daily by mixing 300 mM acetate buffer (pH 3.6), 20 mM ferric chloride, and 10 mM 2,4,6-tripyridyl-*S*-triazine in 40 mM HCl in the ratio of 10:1:1 (*v*/*v*/*v*). The extracted samples (20 μL) were added to 2.8 mL of the FRAP working solution incubated at 37 °C and vortexed. The absorbance was then recorded at 593 nm using a UV-Vis spectrophotometer (UV-2800) after the mixture had been incubated in at 37 °C for 10 min. FRAP values were calculated from FeSO_4_·7H_2_O standard curves and expressed as mmol FeSO_4_·7H_2_O equivalents/μg of film.

### 3.9. Antimicrobial Activities of the Chitosan Films

The agar diffusion method was used to determine the antibacterial activity according to the method of Wang *et al*. [38]. The films were prepared as the above film preparation method. The nutrient agar medium in Petri dish was inoculated with 0.1 mL 10^5^–10^6^ cuf/mL bacteria. The prepared films were cut into 10 mm diameter disks using a hole-puncher and then placed on microbial cultures. Bacterial strains were incubated at 37 °C and 50% ± 2% relative humidity for 24 h. The diameter of the zone of inhibition was measured using a caliper. The tests were performed in triplicate.

### 3.10. Statistical Analysis

Statistical analysis was performed using the SPSS package program version 11.5 (SPSS Inc., Chicago, IL, USA). Data was analyzed by one-way ANOVA, followed by Turkey’s HSD multiple comparison test. The values are reported as means with their standard error for all results. Differences were considered significant at *p* < 0.05.

## 4. Conclusions

The results presented in this study indicate that incorporation of carvacrol, PPE and carvacrol + PPE into chitosan films decreased the transparency, but significantly increased the total phenol content and antioxidant activity. Incorporation of carvacrol and carvacrol + PPE into the films significantly decreased the water vapor permeability, tensile strength and percentage of elongation at break. All the films, with the exception of PPE-incorporated film, exhibited antibacterial activity against *E. coli and S. aureus*. In addition, an additive effect in the antioxidant activity and a synergistic action in the antibacterial activity against the *S. aureus* were found in the film in combined incorporation of carvacrol and PPE. These results suggest the benefits of incorporation of PPE and carvacrol into chitosan films and the potential applications of developed film as an active packaging.
